# Methods to Evaluate the Effects of Internet-Based Digital Health Interventions for Citizens: Systematic Review of Reviews

**DOI:** 10.2196/10202

**Published:** 2018-06-07

**Authors:** Paolo Zanaboni, Patrice Ngangue, Gisele Irène Claudine Mbemba, Thomas Roger Schopf, Trine Strand Bergmo, Marie-Pierre Gagnon

**Affiliations:** ^1^ Norwegian Centre for E-health Research University Hospital of North Norway Tromsø Norway; ^2^ Department of Health Sciences Université du Québec en Abitibi-Témiscamingue Rouyn Noranda, QC Canada; ^3^ Research Center of the CHU de Québec-Université Laval Québec, QC Canada; ^4^ Faculty of Nursing Sciences Université Laval Québec, QC Canada

**Keywords:** review, electronic health records, patient access to records, patient portals, epidemiological methods, evaluation studies as topic

## Abstract

**Background:**

Digital health can empower citizens to manage their health and address health care system problems including poor access, uncoordinated care and increasing costs. Digital health interventions are typically complex interventions. Therefore, evaluations present methodological challenges.

**Objective:**

The objective of this study was to provide a systematic overview of the methods used to evaluate the effects of internet-based digital health interventions for citizens. Three research questions were addressed to explore methods regarding approaches (study design), effects and indicators.

**Methods:**

We conducted a systematic review of reviews of the methods used to measure the effects of internet-based digital health interventions for citizens. The protocol was developed a priori according to Preferred Reporting Items for Systematic review and Meta-Analysis Protocols and the Cochrane Collaboration methodology for overviews of reviews. Qualitative, mixed-method, and quantitative reviews published in English or French from January 2010 to October 2016 were included. We searched for published reviews in PubMed, EMBASE, The Cochrane Database of Systematic Reviews, CINHAL and Epistemonikos. We categorized the findings based on a thematic analysis of the reviews structured around study designs, indicators, types of interventions, effects and perspectives.

**Results:**

A total of 20 unique reviews were included. The most common digital health interventions for citizens were patient portals and patients' access to electronic health records, covered by 10/20 (50%) and 6/20 (30%) reviews, respectively. Quantitative approaches to study design included observational study (15/20 reviews, 75%), randomized controlled trial (13/20 reviews, 65%), quasi-experimental design (9/20 reviews, 45%), and pre-post studies (6/20 reviews, 30%). Qualitative studies or mixed methods were reported in 13/20 (65%) reviews. Five main categories of effects were identified: (1) health and clinical outcomes, (2) psychological and behavioral outcomes, (3) health care utilization, (4) system adoption and use, and (5) system attributes. Health and clinical outcomes were measured with both general indicators and disease-specific indicators and reported in 11/20 (55%) reviews. Patient-provider communication and patient satisfaction were the most investigated psychological and behavioral outcomes, reported in 13/20 (65%) and 12/20 (60%) reviews, respectively. Evaluation of health care utilization was included in 8/20 (40%) reviews, most of which focused on the economic effects on the health care system.

**Conclusions:**

Although observational studies and surveys have provided evidence of benefits and satisfaction for patients, there is still little reliable evidence from randomized controlled trials of improved health outcomes. Future evaluations of digital health interventions for citizens should focus on specific populations or chronic conditions which are more likely to achieve clinically meaningful benefits and use high-quality approaches such as randomized controlled trials. Implementation research methods should also be considered. We identified a wide range of effects and indicators, most of which focused on patients as main end users. Implications for providers and the health system should also be included in evaluations or monitoring of digital health interventions.

## Introduction

### Background

Digital health is defined as the use of digital technologies to provide practical, cost-effective, safe, and scalable interventions to improve health [1], health care services, and wellness for individuals and across populations [2]. Today, numerous types of digital health interventions are available to citizens, patients, carers and the public. They are used to address health system problems including poor access, uncoordinated care and increasingly costly health care [3]. Patient portals [4], mobile health applications [5] and patients’ access to electronic health records (EHR) are common examples of digital health interventions. Interventions can also include other online platforms [6] such as medication refills, appointment scheduling, access to general medical information, or secure messaging between a patient and an institution [7]. Digital health interventions can disseminate information, aid informed decision making, and promote health. They also provide a means for information exchange and support, and manage demand for health services, lowering direct medical costs [8].

Recently, there has been an increasing public interest in digital health [9]. Digital health interventions can empower citizens to track, manage, and improve their health and quality of life while providing a more personalized health care delivery, at a lower cost and with higher efficiency and availability [10]. Digital health has also enabled unprecedented patient engagement in self-management and well-being [2]. Patients seek information from the internet to learn more about their symptoms, diagnoses, and treatments and use a range of digital health interventions to manage their illness at home and support independent living and self-care [11].

Digital health interventions have enormous potential as scalable tools to support better health and health care delivery by improving many different outcomes such as effectiveness, efficiency, accessibility, safety, and personalization [1]. Although evidence of the potential benefit of digital health for improving care delivery and patient outcomes has been described [9], numerous factors can affect patient and public engagement in using digital health interventions such as lack of motivation, busy lifestyle, poor digital literacy, complexity and usability [3]. Other difficulties include the rapid change of technology, which requires digital health interventions to evolve and be constantly updated [1].

Digital health interventions are typically complex with multiple components, and many have multiple aims. As a consequence, evaluations of digital health interventions present unique methodological challenges [1]. A variety of study designs have been implemented in practice to evaluate digital health interventions. Much of the evidence has been generated through quantitative methods, such as pilot studies or clinical trials, although the number of qualitative studies is also increasing [3]. Randomized controlled trials (RCTs) are considered the gold standard in evaluating health care interventions [12]. While RCTs are more likely to be used to assess new treatments or medicines, their applicability to evaluate the complex, multifaceted nature of digital health interventions has been widely debated [13]. RCTs have predefined protocols and strict inclusion criteria that can often mask wider implementation issues [14]. Many challenges only emerge when technologies are scaled up and implemented in ‘real-world’ complex health systems [15]. When evaluating a digital health intervention, it is essential to identify the likely benefits, define the causal model describing how the intervention will achieve its intended benefits, and broaden the portfolio of evaluation methods [1].

Researchers need to support the public, patients, clinicians, and policy-makers by creating an actionable knowledge base to identify the effects of digital health. Specific frameworks for evaluating digital health interventions have been recently developed to generate evidence required for decision-making on the appropriate approach to integrate effective strategies into broader national health systems [16]. An example is the Canada Health Infoway Benefits Evaluation Framework based on dimensions of quality, system usage and net benefits, as well as specific indicators [17]. Careful monitoring and systematic evaluations of digital health interventions, however, have been few, in contrast to the proliferation of digital health pilot projects [16]. As a consequence, the current research evidence on which methods should be used to evaluate digital health interventions is still fragmented [3].

The objective of the current study was to explore and provide a systematic overview of the methods used to evaluate the effects of internet-based digital health interventions for citizens. Internet-based digital health interventions covered by this study included patients’ access to EHR, patient portals, and other internet-based support programs. Citizens are referred to as the general population, including both healthy individuals and patients having access to health care services.

The following research questions were addressed:

1. Which approaches (study design) have been used to produce knowledge about the effects of internet-based digital health interventions for citizens?
2. Which effects have been measured and reported in studies focusing on internet-based digital health interventions for citizens?
3. Which indicators were used to measure the effects of internet-based digital health interventions for citizens, and for whom were the effects measured (patients, health care system, society)?

## Methods

### Study Design

A systematic review of reviews [18] focused on the methods used to measure the effects of internet-based digital health interventions for citizens was conducted in accordance with the Preferred Reporting Items for Systematic review and Meta-Analysis Protocols (PRISMA-P) [19]. This includes a checklist of recommended items to be addressed in a systematic review protocol [20]. The protocol was developed a priori according to the International Prospective Register of Systematic Reviews (PROSPERO) template and is available upon request. We followed the Cochrane Collaboration methodology for overviews of reviews [21]. The scope of the review and eligibility criteria were formulated using the PICOS approach (participants, interventions, comparisons, outcomes, study designs) [21]. The Assessing the Methodological Quality of Systematic Reviews (AMSTAR) checklist was used to assess the quality of the included reviews [22].

### Inclusion and Exclusion Criteria

The systematic review of reviews included qualitative, mixed-method, and quantitative studies published in English or French from January 2010 to October 2016. Review papers were eligible if (1) the primary end-user was the patient or carer or citizen, (2) they were related to internet-based digital health interventions for citizens where there was a direct form of interaction with health care providers, and (3) they evaluated the impact of implementing or using internet-based digital health interventions for citizens. Internet-based digital health interventions for citizens included patients’ access to EHR, patient portals, and other internet-based support programs. An EHR is the electronic collection of clinical data relating to one subject of care, including clinical assessments, laboratory results, radiology findings, nursing documentation, allergy information, medication information and discharge letters. Health care organizations can provide online EHR access to patients, relatives or other informal carers. Besides health care organizations, EHR access may also be offered on a national scale [7]. Electronic patient portals are defined as electronic applications (typically web-based) provided and maintained by health care institutions which can offer access to (a subset of) clinical EHR data as well as additional services, including medication refills, appointment scheduling, access to general medical information such as guidelines, or secure messaging [7]. Internet-based support programs refer to interventions, such as social support groups, online therapy for psychosocial or physical symptoms, online systems integrating information, support and coaching services [23], which can promote collaboration and help individuals with chronic conditions. Review papers were excluded if (1) they were related to interventions used solely by health care professionals (eg, clinical decision support systems), (2) they were related to interventions designed for patients without direct interaction with health care providers (eg, mHealth, self-management tools, educational platforms), and (3) they were focused on patient access to health records which were not digital (eg, paper records).

### Data Sources and Search Strategy

We searched for reviews published in the following electronic bibliographic databases: PubMed, EMBASE, The Cochrane Database of Systematic Reviews, CINHAL and Epistemonikos. A structured search strategy was developed using the thesaurus terms of each database and using some keywords included in the titles and abstracts of the reviews. The search strategy included terms relating to or describing internet-based digital health interventions for citizens (Multimedia Appendix 1). The results of each database search were stored in a single reference database (Endnote). Duplicate references were removed. The electronic search on the mentioned databases was performed by one research team member (PN).

### Study Selection

Titles and abstracts of the review papers retrieved using the search strategy were screened by two reviewers (PN, GM). Studies that did not meet the inclusion criteria were excluded. The full texts of the selected studies were then retrieved and independently assessed for eligibility by a review team consisting of six members (PZ, TRS, TB, MPG, PN, GM). Any disagreement over the eligibility of particular studies was resolved through discussion and the involvement of another reviewer if necessary.

### Data Extraction

A standardized data extraction form was developed, piloted and used to extract data from the full text of the included reviews for evidence synthesis (Multimedia Appendix 2). Extracted data used to categorize review papers included first author, year of publication, language, type of review, rationale, objectives, eligibility criteria, and the fields of the AMSTAR checklist. Additional information extracted from each review paper included: study selection, interventions, populations, settings, effects measured (types of outcome, perspectives, indicators), study design, and main findings.

Three papers were chosen to pilot the data extraction process and form. All the review team members reviewed them. A meeting was organized around data extraction to make sure that all review team members had the same understanding of the information to extract.

The full texts of the included reviews were equally and randomly divided among the review members to minimize bias. For each paper, data were extracted systematically and reported on the data extraction form. Papers that did not meet the inclusion criteria were pointed out along with the reasons for exclusion. Another member then cross-checked all the papers reviewed by one member in order to agree on the selection.

### Quality Assessment

The AMSTAR checklist was used to assess the quality of the included studies [22]. AMSTAR is an 11-item checklist from which reviewers assign 1 point when 1 criterion is met. This tool characterizes the quality of a systematic review at 3 levels: a score 8-11 is considered high quality (ie, minor or no methodological limitations), a score 4-7 is medium quality (ie, moderate methodological limitations), while a score 0-3 is low quality (ie, significant methodological limitations). Because this study was aimed at describing and synthesizing a body of both quantitative and qualitative literature, and not determining an effect size, no additional methods for risk of bias were conducted as they would not have affected the interpretative synthesis of the findings.

### Data Synthesis

We conducted a thematic analysis of the outcomes of the included papers. Thematic analysis is the most common method adopted within narrative reviews to produce a synthesis of findings arising from a body of literature. It seeks to identify systematically emerging conceptual themes across multiple studies. The themes identified are shaped by the specific review questions [24]. The findings were analysed and structured around the study designs and indicators used to measure the effects of different interventions, the types of interventions, the impact of interventions and the perspectives for whom the outcomes were measured to answer the three research questions. Additional information was also extracted and analyzed to describe the evidence and direction of the effects reported in the included papers. The results were summarized in the form of a textual, narrative understanding of the findings supported by tabular summaries.

## Results

### Search Results

**Figure 1:**
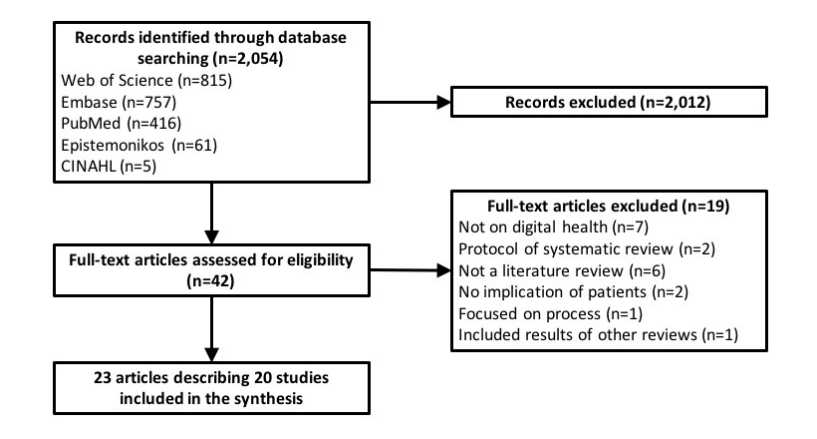
Preferred Reporting Items for Systematic review and Meta-Analysis (PRISMA) study flow diagram.

A total of 2,054 papers were identified from the search strategy. After removing duplicates and initial screening of titles and abstracts, 42 articles were retrieved for detailed evaluation. Following further inspection of their full-texts, 23 papers met the eligibility criteria (Multimedia Appendix 3), while the remaining 19 articles were excluded (Multimedia Appendix 4). Of the 23 papers included, there were 3 publications [25,26,27] which were part of another main study [7,28,29], leading to a total of 20 unique reviews [4,7,23,28-44]. The overall process of review selection was summarized according to the PRISMA study flow diagram (Figure 1).

### Review Characteristics

The general characteristics of the studies, including the type of review [45], population, intervention, setting, and quality, were summarized by the information extracted from the analysis of the full-text articles (Table 1). There were 14/20 (70%) systematic reviews and 1/20 (5%) systematic review which also included a meta-analysis. Among the remaining reviews, 3/20 (15%) were literature, 1/20 (5%) was realist, and 1/20 (5%) was narrative. The most common internet-based digital health interventions for citizens included in the present study were patient portals and patients’ access to their EHR, covered by 10/20 (50%) and 6/20 (30%) reviews, respectively. Internet-based support programs were described in 4/20 (20%) studies. A total of 8/20 (40%) did not have any restriction regarding the study settings. Where the setting was specified, 6/20 (30%) reviews were focused on primary care, while the remaining (6/20, 30%) included outpatient and inpatient settings. According to the AMSTAR score 13/20 (65%) reviews were medium quality, while 6/20 (30%) were high quality. Only 1/20 (5%) review scored low quality. Systematic reviews had on average a higher quality than other types.

**Table 1:** Characteristics of included studies.

Reference	Year	Studies, n	Type of review	Population	Intervention	Setting	AMSTAR^a^
Alkureishi et al [30]	2016	53	Systematic review	Adult and paediatric patients	Patients’ access to EHR^b^	Outpatient and inpatient	7
Amante et al [31]	2014	16	Systematic review	Patients with diabetes	Patient portals	Primary care	8
Ammenwerth et al [7]	2012	5	Systematic review	Patients	Patient portals	Outpatient and inpatient	8
Bouma et al [23]	2015	16	Literature review	Cancer patients	Internet-based support programs	No restrictions	8
Bush et al [32]	2016	31	Systematic review	Paediatric patients	Patient portals	No restrictions	4
Davis Giardina et al [33]	2014	27	Systematic review	Patients	Patients’ access to EHR	No restrictions	5
Davis et al [34]	2014	16	Systematic review	Adult patients	Internet-based support programs	Primary care	6
Goldzweig et al [35]	2013	46	Systematic review	Patients	Patient portals	Primary care	8
Irizarry et al [4]	2014	120	Literature review	Patients	Patient portals	No restrictions	6
Kruse et al [29]	2015	27	Systematic review	Patients with chronic conditions	Patient portals	Outpatient and inpatient	4
Liu et al [36]	2013	8	Systematic review	Patients	Patients’ access to EHR	Primary care, emergency, outpatient	6
Mold et al [28]	2015	17	Systematic review	Patients	Patients’ access to EHR	Primary care	9
Osborn et al [37]	2010	26	Systematic review	Patients with diabetes	Patient portals	Primary care	6
Otte-Trojel et al [38]	2014	32	Realist review	Patients	Patient portals	Outpatient and inpatient	4
Price et al [39]	2015	23	Systematic review	Patients with chronic conditions	Patients’ access to EHR	Outpatient	5
Stellefson et al [40]	2013	15	Systematic review	Patients with chronic conditions	Internet-based support programs	No restrictions	7
Tao and Or [41]	2013	36	Meta-analysis	Patients with diabetes	Internet-based support programs	No restrictions	10
Tulu et al [42]	2016	23	Literature review	Patients with pulmonary conditions	Patient portals	Pulmonary practice	4
Turner et al [43]	2016	12	Narrative review	Patients with HIV	Patients’ access to EHR	No restrictions	3
Vimalananda et al [44]	2015	27	Systematic review	Patients, specialty care	Patient portals	No restrictions	6

^a^AMSTAR: Assessing the Methodological Quality of Systematic Reviews. Studies are classified as high (scoring 8-11), medium (4-7), or low quality (0-3).

^b^EHR: electronic health record.

### Overview of Research Methods

Extracted data from the studies included in this review were then analysed to address the first research question by providing an overview of the types of study design used to measure the effects of different internet-based digital health interventions for citizens (Table 2). Each review could be focused on one type of study only (eg, RCT) or include different study designs. Overall, 13/20 (65%) reviews summarised results from both quantitative and qualitative studies (including mixed methods), while the remaining 7/20 (35%) reviews contained only studies using quantitative methods (RCTs, quasi-experimental studies with control, cohort, pre-post studies, retrospective studies, cross-sectional studies, and surveys).

A total of 13/20 (65%) of the included reviews reported the use of RCTs to evaluate patients’ access to EHR, patient portals, and internet-based support programs. Quasi-experimental designs, in form of non-randomized controlled trials where subjects are allocated to intervention and control groups without a randomization method, were described in 9/20 (45%) reviews. Another method, which does not imply randomization and does not necessarily require a control group, is a pre-post study (or before-after study). Pre-post studies measure a specific outcome before and after an intervention. However, due to the lack of a control group, this study design is considered weak as it is difficult to conclude whether changes occurred due to the intervention or would have occurred anyway. Pre-post studies were reported only in 6/20 (30%) reviews.

**Table 2:** Overview of research methods. No data is shown as N/A (not applicable).

Study design	Interventions		
	Patients’ access to EHR^a^	Patient portals	Internet-based support programs
RCT^b^	[28,33,36,39]	[4,7,31,35,37,38]	[23,40,41]
Quasi experimental with control	[28,43]	[4,29,35,37,44]	[23,40]
Pre-post	[29,33,36]	[35,37,44]	N/A^c^
Cohort	[28,33,39]	[4,31,38,44]	N/A
Retrospective	[33,39]	[29,33,44]	N/A
Cross-sectional or surveys	[28,30,33,36,39,43]	[4,29,31,32,35,42,44]	[34,40]
Qualitative	[30,43]	[4,29,31,32,35,37,38,42,44]	[34,40]
Mixed methods	[30,43]	[29,31,37,38]	[34]
Other (pilot study, simulation, usability)	N/A	[4,32,37,44]	N/A

^a^EHR: electronic health record.

^b^RCT: randomized controlled trial.

^c^N/A: not applicable.

As an alternative to experimental and quasi-experimental designs, 15/20 (75%) reviews reported using observational studies to evaluate internet-based digital health interventions for citizens. These include prospective cohort studies, retrospective studies, cross-sectional studies, and surveys. Observational studies were reported for all the types of digital health interventions included in this review.

Finally, 13/20 (65%) reviews included evaluation of internet-based digital health interventions through qualitative studies or mixed methods. Use of qualitative methods was described in all the types of digital health interventions covered by this review. Some (4/20, 20%) reviews also referred to the use of other research methods including pilot studies, simulation or usability testing.

### Overview of Effects and Indicators

To address the second and third research questions, we extracted and analyzed study data regarding which effects were measured and reported, which methods (regarding indicators) were used to measure the effects, and for whom they were measured (patients, providers, health care system, or society). These data are summarized in Table 3.

A large number of effects were measured when evaluating internet-based digital health interventions for citizens. These were classified into five main categories (1) health and clinical outcomes, (2) psychological and behavioral outcomes, (3) health care utilization, (4) system adoption and use, and (5) system attributes. The first two categories are mainly related to effects perceived by patients as main end users. While there was limited evidence of the clinical benefits for patients resulting from the implementation of internet-based digital health interventions, such as access to their EHR or patient portals, health and behavioral outcomes were often included as an object of evaluation. Health care utilization refers to a range of indicators which can affect both patients and the health care system in general. Adoption and use is another critical category related to the users of digital health interventions, namely patients and providers. Finally, system attributes refer to other effects focusing on the evaluation of the systems themselves, which can impact on patients, providers, and the health system in general.

Health and clinical outcomes were measured with both general indicators (eg, improvement in health status, quality of life, medication management, mortality, physical activity) and disease-specific indicators (eg, related to diabetes or hypertension). Overall, there were 11/20 (55%) studies which reported health and clinical outcomes.

Psychological and behavioral outcomes include a wide variety of indicators reflecting the impact on patients in changing their behavior towards the way they manage their health or a specific disease. Patient-provider communication was by far the most investigated indicator. Internet-based digital health interventions are claimed to impact on the quality of the communication between patients and health providers, and 13/20 (65%) studies examined this effect. Satisfaction was another widely used indicator. Patient satisfaction was documented in 12/20 (60%) reviews, while only 1/20 (5%) review additionally reported satisfaction for providers. Self-efficacy and self-management represent other important indicators which were included in 5/20 (25%) reviews. However, similarly to clinical outcomes, evidence of the effects on self-efficacy is limited. Other psychological and behavioral outcomes included: adherence to therapy, potential harms, perceived benefits, and perceived social support. Specific indicators measuring a change in the role of patients towards health services were: improved access to information, attitudes, empowerment, acceptance, and endorsement.

**Table 3:** Overview of effects and indicators. No data is shown as N/A (not applicable).

Outcomes and indicators		Perspective			
		Patients	Providers	Health system	Society
**Health and clinical outcomes**					
	Health status	[29,35,37,38,40,44]	N/A^a^	N/A	N/A
	Quality of life	[23,39,40]	N/A	N/A	N/A
	Safety or medication management	[28,39]	N/A	N/A	N/A
	Disease-specific measures	[32,39,41]	N/A	N/A	N/A
	Mortality or risk factors	[7]	N/A	N/A	N/A
	Physical activity or nutrition outcomes	[40]	N/A	N/A	N/A
**Psychological and behavioral outcomes**					
	Self-management or self-efficacy	[33,39,40,42,43]	N/A	N/A	N/A
	Satisfaction	[7,28-30,33,35-39,44]	[34]	N/A	N/A
	Patient activation	[39]	N/A	N/A	N/A
	Patient-provider communication	[4,7,28-30,33,37,38,42,43]	[4,30,37]	[31,34]	N/A
	Patient access to information	[39]	N/A	N/A	N/A
	Acceptance or endorsement	N/A	[4,44]	N/A	N/A
	Health literacy	[4,37]	N/A	N/A	N/A
	Awareness and knowledge	[39,43]	N/A	N/A	N/A
	Perceived benefits	[28,33,43]	N/A	N/A	N/A
	Concerns (privacy, security)	[28,43]	N/A	N/A	N/A
	Perceived social support	[23,40]	N/A	N/A	N/A
	Adherence to treatment	[7,31,33,35,38,43]	N/A	N/A	N/A
	Empowerment	[7,29,31,37,38]	N/A	N/A	N/A
	Attitudes	[35]	[4]	N/A	N/A
	Harms (distress, stress, anxiety)	[23,33,39,40]	N/A	N/A	N/A
**Health care utilization**					
	Outpatient or clinic visits	[28]	N/A	[7,33,38,39,44]	N/A
	Access or wait time	[28,42,44]	N/A	N/A	N/A
	Hospitalization rate or urgent care utilization	[7]	N/A	[7,29,33,40]	N/A
**System adoption and use**					
	Patient adoption	[4,32]	N/A	N/A	N/A
	Professional practice	N/A	[31,34]	N/A	N/A
	Patient utilization	[33,40]	N/A	N/A	N/A
**System attributes**					
	Usability	[4,32,37,38,42,43]	[4,38]	N/A	N/A
	Utility	[4]	N/A	N/A	N/A
	Personalization	[4]	N/A	N/A	N/A
	Efficiency	[35]	N/A	[34]	N/A

^a^N/A: not applicable.

Health care utilization refers to the impact of digital health interventions on the resources involved, including time used by patients and providers and use of the health care system (eg, hospitalizations and outpatient care). Evaluation of health care utilization was included in 8/20 (40%) reviews, most of which focused on the economic effects on the health care system.

System adoption and use was measured through three indicators, two of which were defined from the patient’s perspective. Patient adoption and patient utilization refer to how patients decide to use a digital health intervention and to what extent this is used in practice. Professional practice indicates the degree to which a digital health service implies organizational changes for health care professionals. Each of these indicators was covered by 2/20 (10%) reviews.

Other indicators were also used to measure different system attributes. Of these, the most relevant was usability for patients and providers, which was included in 6/20 (30%) reviews. Each of the indicators was measured with specific tools, including data recorded from patients, questionnaires, data collected from databases or registries, and interviews.

### Synthesis of the Evidence of Benefits

We finally summarized the main findings regarding the outcomes of different internet-based digital health interventions included in this review to provide a narrative description of the evidence of benefits. A total of 3/6 (50%) reviews focusing on the effects of providing patients access to their EHR found improved levels of patient satisfaction [28,33,36]. However, evidence was less clear for effects on health care quality, including measures of safety, effectiveness, patient-centeredness, timeliness, efficiency, and equity [33]. There have been only a few RCTs examining the effectiveness of online access to EHR on improving health outcomes, but some work has been undertaken focusing on the impact on patient decision making and health outcomes [28]. Patients reported some evidence of improvements in safety through identifying medication errors, self-care, communication and engagement with clinicians [28]. There were no reports of harm to patients, such as increased anxiety, a common fear endorsed by physicians [33], nor breaches in privacy [28]. However, some participants were concerned about privacy and security [43]. Effects on workload and system efficiency were unclear [28,33]. Evidence of benefits, including quality of care, access, productivity, self-management, associated with the use of EHR by citizens were found for patients with specific chronic health conditions [39]. In general, low use was associated with socio-demographic factors [43].

Evidence that patient portals improve health outcomes was mixed [35]. There were 3/10 (30%) reviews reporting positive effects of patient portals on better adherence to treatment and medication regimen [7,29,38]. However, overall evidence of improvement in quality of care was limited [**7**], and there was a lack of studies investigating long-term health outcomes [32]. As most of the patient portal evaluations targeted online interventions for chronic disease management (eg, diabetes, hypertension, depression, chronic musculoskeletal pain, or mobility difficulty), evidence of benefits was found for disease awareness and self-efficacy [29,37,41], empowerment [29,38], patient-provider communication and social support [37,38,40]. There was some evidence that patient portals impact on costs or utilization [35], including a decrease in office visits [7,29]. However, there were also some studies which found a higher health resource use [38]. In general, there was a high satisfaction and acceptance by users of patient portals [29,32,37,38,42]. Facilitators to adoption and use of patient portals were encouragement and assistance from providers and family members as well as self-engagement, while barriers included lack of skills, desire, or knowledge, technical difficulties, and lack of potential benefits [31]. Patient engagement and use of patient portals was strongly influenced by personal factors (ethnicity, education level, health literacy, health status) and health care delivery factors (provider endorsement and usability) [4]. Patient portals were mainly used by patients with chronic conditions [32]. No studies found serious adverse consequences [38].

Similarly to patient portals, other internet-based support programs for patients with chronic conditions found evidence of benefits regarding increased self-efficacy and better communication with the health care providers [40]. There was 1/4 (25%) review focused on internet support programs for cancer patients that found some positive results on quality of life and social support, as well as on disease-specific indicators including cancer-related fatigue, insomnia, and stress [23]. A meta-analysis of RCTs showed that the use of health information technology was associated with an improved glycaemic control in patients with diabetes [41].

## Discussion

### Principal Findings

This systematic review provides an overview of the types of study design and methods used to measure the effects of internet-based digital health interventions for citizens which are reported in other reviews. We found 20 relevant reviews published since 2010, indicating a generally growing interest in the evaluation of the effects of digital health interventions for citizens such as patients’ access to their EHR and patient portals. The overall quality of the included reviews was also good, with many high-quality studies and only a few studies with methodological limitations. Non-systematic reviews generally had a lower quality score on the AMSTAR scale because this tool was specifically designed to assess systematic reviews of quantitative studies, and there is no equivalent tool for other types of reviews [46].

Although there is some evidence of benefits from observational studies and surveys, with many studies reporting value in patients having access to more information through internet-based digital health interventions, there is still little reliable evidence from experimental studies of proven effectiveness in improved patient health outcomes [47]. One reason for this may be that not all health conditions are sensitive to patients’ access to EHR as an intervention [39]. Conditions with evidence of clinical benefits for patients accessing EHR include chronic diseases (eg, diabetes) with an aspect of monitoring, either by the clinician or the patient (self-monitoring). RCTs are needed to test assumptions about the comparative effectiveness on outcomes for various patient populations [47]. However, their applicability to evaluate the complex multifaceted nature of digital health interventions has been widely debated [13]. RCTs remain an essential method for determining the impact of digital health interventions concerning efficacy and cost-effectiveness but are best undertaken when the services are highly likely to lead to clinically meaningful benefits [1]. As a consequence, patient portals, online services or internet support programs specifically designed for chronic disease management seem to be more suitable to the use of RCTs as a research method to measure the effects on clinical outcomes. In case of digital health interventions offered to citizens, RCTs might also be useful to measure the impact on those sub-populations of people who are more likely to obtain improved outcomes.

RCTs are also best undertaken once the services are stable and can be implemented with high fidelity [1]. However, digital health interventions are typically complex interventions with multiple components and multiple aims [1]. As a consequence, evaluations present unique methodological challenges. The successful development, integration, and implementation of digital health interventions require a radical shift from traditional, and single-disciplinary academic and clinical approaches [2]. In this respect, the Normalisation Process Theory addresses the factors needed for successful implementation and integration of interventions into routine work, enabling researchers to think through issues of implementation while designing a complex intervention and its evaluation. [48]. Implementation research is another growing field which seeks to understand and work within real-world conditions, rather than trying to control for these conditions or to remove their influence as causal effects [49]. Implementation research provides a framework for using the research question as the basis for selecting among a wide range of qualitative and quantitative methods. Implementation specific research methods include non-traditional studies such as pragmatic trials, effectiveness-implementation hybrid trials, quality improvement studies, participatory action research, and mixed methods [49]. The types of study design used to evaluate internet-based digital health interventions were experimental studies, observational studies, and qualitative studies. We found only a few mixed methods studies in our review, but no other types of implementation research methods were reported. Future evaluations of internet-based digital health interventions should be appropriately designed, and where RCTs are not appropriate, make more use of implementation research methods.

The studies included in this review allowed identifying a wide range of effects and indicators, which can affect patients, providers and the health system in general. These methods can be used in evaluations of new internet-based digital health interventions implemented in the future or to monitor them over time after their introduction. Most indicators were focused on measuring direct impact on the patients as main end users. However, evaluations can also benefit from including indicators used to measure the effects for providers (such as acceptance, communication, and usability) and the health system (such as health care utilization). There is insufficient use of indicators that measure the impact on society, indicating that societal effects have minor importance, or are simply more difficult to measure.

The high number of studies measuring satisfaction and acceptance by users suggests that internet-based digital health interventions might have an important impact on patients in changing their behavior towards the way they manage their health or a specific disease. One reason might be that digital health interventions designed and implemented to improve health services for citizens/patients are more likely to have an impact on their level of satisfaction. Another reason might be that satisfaction is an indicator relatively easy to measure and monitor over time.

Measuring system adoption and use are vital to understand how patients and providers decide to adopt internet-based digital health interventions and implement them in practice. These indicators are relevant to monitor the overall impact of digital health interventions (ie, scalability) over time. People with serious chronic conditions, individuals with disabilities, parents with small children, people with a keen interest in maintaining healthy lifestyles, and the elderly or their caregivers seem more likely to adopt internet-based digital health interventions [47]. System usability for both patients and providers can in turn impact on satisfaction and use and must, therefore, be included in evaluation and monitoring activities. Patients’ interest and ability to use digital health interventions is strongly influenced by personal factors such age, ethnicity, education level, health literacy, health status, and role as a caregiver [35,37,43]. Future research should focus on identifying specific characteristics associated with a higher degree of patient engagement [4].

Evidence of clinical outcomes was still unclear, for both patients’ access to EHR and patient portals. There were, however, some positive results for improved health status and better medication management. Several studies included measurement of psychological and behavioral indicators, with evidence that internet-based digital health interventions can improve self-efficacy. Effects were more significant for patients with specific chronic health conditions. Additional research is needed to identify which features are most influential in changing health behaviors [43].

Effects on workload and system efficiency were also unclear, with some studies reporting savings, while other studies reported increased health service use when using patient portals. This suggests that patient portals might be seen as complements rather than substitutes to existing health services [38]. The inclusion of cost-effectiveness evaluations, or simpler indicators to evaluate the impact of internet-based digital health interventions on health care utilization, is necessary as the economic impact can represent an incentive for both patients and providers to use the systems.

### Study Strengths and Limitations

The findings of the current study emerge from the analysis of reviews focused on the evaluation of the effects of different internet-based digital health interventions for citizens, rather than from the analysis of individual studies. Moreover, the results were summarised in the form of a thematic analysis since a meta-analysis was not possible due to the heterogeneity of the included reviews. However, the objective of this study was to explore and provide a systematic overview of the methods used to evaluate the effects of internet-based digital health interventions for citizens. That is, we did not intend to provide a quantitative synthesis of the effects. As such, we chose to conduct a systematic review of reviews which allowed us to answer our research questions. An a priori protocol was developed per international quality standards including the Cochrane Collaboration methodology for overviews of reviews, PRISMA-P and PROSPERO templates. The scope and eligibility criteria were formulated using the PICOS approach. Moreover, most of the reviews included in this review were high-quality studies according to the AMSTAR checklist. Although our research did not aim at quantifying the outcomes reported in the included papers, we were still able to describe the primary evidence and direction of the effects.

Another limitation of this study was that it was not focused on a specific intervention. On the contrary, it aimed to gather information on which methods were used in the evaluation of digital health interventions. As a consequence, we purposefully chose to include reviews which were focused on a wide range of internet-based digital health interventions. This allowed us to provide a complete picture of which methods were used across different services and identify similarities or specificities. Moreover, mobile health (mHealth) interventions were beyond the scope of this study and thus not explicitly covered in the review. The term mHealth refers to the use of mobile and wireless technologies for health [50] and includes interventions that are delivered through mobile devices or the new generation of tablet computers [51].

The study protocol was designed to include only studies published from 2010. Reviews published earlier were therefore excluded. Digital health is a relatively recent field, and the majority of reviews focusing on digital health interventions has been published over the past few years. As we might have missed a few studies published before 2010, those studies are likely to be included in recent reviews. Moreover, as digital health technologies are always in rapid change, it appeared to be relevant to focus on studies which were recently conducted. The terms included in the search strategy might have also been restrictive when referring to citizens and patients. Despite these limitations, more than 2,000 studies were screened, and a total of 23 publications involving 20 unique reviews were included in this review.

### Conclusions

We found many relevant reviews, indicating a generally growing interest in the evaluation of the effects of internet-based digital health interventions such as patients’ access to EHR and patient portals. Although there is some evidence of benefits and satisfaction for patients from observational studies and surveys, there is still little reliable evidence from RCTs or other experimental studies of proven effectiveness in improved patient health outcomes through the use of digital health interventions. The results of this review show that internet-based digital health interventions for citizens have a higher clinical impact on chronic disease management. Future evaluation studies focused on clinical outcomes should possibly focus on specific populations or chronic conditions which are more likely to achieve clinically meaningful benefits and use high-quality methods for study design, such as RCTs. Researchers should think through issues of implementation while designing and evaluating complex digital health interventions. Moreover, non-traditional approaches such as implementation research methods should be considered as valuable alternatives when evaluating internet-based digital health interventions. Additional research is also needed to identify which personal health record features are most influential in changing health behaviors.

## Appendix

Multimedia Appendix 1 Search strategy.

Multimedia Appendix 2 Data extraction form.

Multimedia Appendix 3 Studies included.

Multimedia Appendix 4 Studies excluded.

## Abbreviations

AMSTAR: Assessing the Methodological Quality of Systematic Reviews
EHR: Electronic Health Records
PICOS: Participants, Interventions, Comparisons, Outcomes, Study Designs
PRISMA-P: Preferred Reporting Items for Systematic review and Meta-Analysis Protocols
PROSPERO: International Prospective Register of Systematic Reviews
RCT: Randomized Controlled Trial
