# Supplementary material for: Methods to Evaluate the Effects of Internet-Based Digital Health Interventions for Citizens: Systematic Review of Reviews
Source: J Med Internet Res. 2018 Jun 7;20(6):e10202. doi: 10.2196/10202 (PMC6013714; doi:10.2196/10202)
Supplement: Multimedia Appendix 1 [file jmir_v20i6e10202_app1.pdf]

TS=(Citizen **OR** citizens **OR** patient **OR** patients **OR** “patient portal*”)

AND

TS=(“health record*” **OR** “electronic health record*” **OR** “medical record*” **OR** “electronic medical record*” **OR** “electronic patient record*” **OR** “online record*” **OR** “online health record*” **OR** “data online” **OR** “personal data” **OR** “personal medical data” **OR** “personal medical record*” **OR** “personal health record*” **OR** “health data” **OR** “medical data” **OR** “personal data” **OR** “digital data” **OR** “digital health data” **OR** “Public eHealth Service” **OR** “online services” **OR** “laboratory results” **OR** “electronic tools” **OR** “electronic patient services” **OR** “health information exchange” **OR** “doctors notes” **OR** “doctors visit notes” **OR** “digital health”)

AND

“patient-provider communication” **OR** “Patient-physician” **OR** interaction* **OR** “e-consultation” **OR** “secure messaging” **OR** “web messaging” **OR** “electronic messaging” **OR** “electronic communication” **OR** “email communication” **OR** “e-mail consultations” **OR** SMS **OR** “Video consultation” **OR** “patient engagement*”
