# Supplementary material for: Methods to Evaluate the Effects of Internet-Based Digital Health Interventions for Citizens: Systematic Review of Reviews
Source: J Med Internet Res. 2018 Jun 7;20(6):e10202. doi: 10.2196/10202 (PMC6013714; doi:10.2196/10202)
Supplement: Multimedia Appendix 2 [file jmir_v20i6e10202_app2.pdf]

Results and outcomes
ID
First author
Year of publication
Study selection: numbers of studies screened/ assessed for eligibility/ included in the review
Intervention(s)
Population(s)
Setting(s) (clinical or other)
Regions or countries in which the included studies were conducted
Type of outcome #01
For who? (patient, healthcare system, society)
Indicators (methods of measurement)
Type of outcome #02
For who? (patient, healthcare system, society)
Indicators (methods of measurement)
Type of outcome #03
For who? (patient, healthcare system, society)
Indicators (methods of measurement)
Type of outcome #04
For who? (patient, healthcare system, society)
Indicators (methods of measurement)
Type of outcome #05
For who? (patient, healthcare system, society)
Indicators (methods of measurement)
Type of outcome #06
For who? (patient, healthcare system, society)
Indicators (methods of measurement)
Type of outcome #07
For who? (patient, healthcare system, society)
Indicators (methods of measurement)
Type of outcome #08
For who? (patient, healthcare system, society)
Indicators (methods of measurement)
Type of outcome #09
For who? (patient, healthcare system, society)
Indicators (methods of measurement)
Type of outcome #10
For who? (patient, healthcare system, society)
Indicators (methods of measurement)
Results of risk of bias (how review authors considered bias in their interpretation of findings)
Study design(s)
Results of each meta-analysis
Main findings for main outcomes (strength of evidence)
Results of additional analysis
Limitations
Conclusion
Comments
