# Supplementary material for: Methods to Evaluate the Effects of Internet-Based Digital Health Interventions for Citizens: Systematic Review of Reviews
Source: J Med Internet Res. 2018 Jun 7;20(6):e10202. doi: 10.2196/10202 (PMC6013714; doi:10.2196/10202)
Supplement: Multimedia Appendix 3 [file jmir_v20i6e10202_app3.pdf]

1. **Alkureishi** MA, Lee WW, Lyons M, Press VG, Imam S, Nkansah-Amankra A, et al. Impact of Electronic Medical Record Use on the Patient-Doctor Relationship and Communication: A Systematic Review. Journal of General Internal Medicine. 2016;31(5):548-60.
2. **Amante** DJ, Hogan TP, Pagoto SL, English TM. A systematic review of electronic portal usage among patients with diabetes. Diabetes Technology and Therapeutics. 2014;16(11):784-93.
3. **Ammenwerth** E, Schnell-Inderst P, Hoerbst A. Patient empowerment by electronic health records: first results of a systematic review on the benefit of patient portals. Studies in health technology and informatics. 2011;165:63-7.
4. **Ammenwerth** E, Schnell-Inderst P, Hoerbst A. The impact of electronic patient portals on patient care: a systematic review of controlled trials. Journal of medical Internet research. 2012;14(6):e162.
5. **Bouma** G, Admiraal JM, de Vries EGE, Schröder CP, Walenkamp AME, Reyners AKL. Internet-based support programs to alleviate psychosocial and physical symptoms in cancer patients: A literature analysis. Critical Reviews in Oncology/Hematology. 2015;95(1):26-37.
6. **Bush** RA, Connelly CD, Fuller M, Perez A. Implementation of the Integrated Electronic Patient Portal in the Pediatric Population: A Systematic Review. Telemedicine and E-Health. 2016;22(2):144-52.
7. **Davis** MM, Freeman M, Kaye J, Vuckovic N, Buckley DI. A systematic review of clinician and staff views on the acceptability of incorporating remote monitoring technology into primary care. Telemed J E Health. 2014;20(5):428-38.
8. **Davis Giardina** T, Menon S, Parrish DE, Sittig DF, Singh H. Patient access to medical records and healthcare outcomes: a systematic review. J Am Med Inform Assoc. 2014;21(4):737-41.
9. **de Lusignan** S, Mold F, Sheikh A, Majeed A, Wyatt JC, Quinn T, et al. Patients' online access to their electronic health records and linked online services: a systematic interpretative review. BMJ Open. 2014;4(9):e006021.
10. **Goldzweig** Dr CL, Orshansky Dr G, Paige Dr NM, Towfigh Dr AA, Haggstrom Dr DA, Miake-Lye Dr I, et al. Electronic patient portals: Evidence on health outcomes, satisfaction, efficiency, and attitudes. Annals of Internal Medicine. 2013;159(10):677-87.
11. **Irizarry** T, Dabbs AD, Curran CR. Patient Portals and Patient Engagement: A State of the Science Review. Journal of medical Internet research. 2015;17(6).
12. **Kruse** CS, Argueta DA, Lopez L, Nair A. Patient and provider attitudes toward the use of patient portals for the management of chronic disease: a systematic review. Journal of medical Internet research. 2015;17(2):e40.
13. **Kruse** CS, Bolton K, Freriks G. The effect of patient portals on quality outcomes and its implications to meaningful use: a systematic review. Journal of medical Internet research. 2015;17(2):e44.
14. **Liu** JL, Luo L, Zhang R, Huang TT. Patient satisfaction with electronic medical/health record: a systematic review. Scandinavian Journal of Caring Sciences. 2013;27(4):785-91.
15. **Mold** F, Ellis B, de Lusignan S, Sheikh A, Wyatt JC, Cavill M, et al. The provision and impact of online patient access to their electronic health records (EHR) and transactional services on the quality and safety of health care: systematic review protocol. Inform Prim Care. 2012;20(4):271-82.
16. **Osborn** CY, Mayberry LS, Mulvaney SA, Hess R. Patient Web Portals to Improve Diabetes Outcomes: A Systematic Review. Current Diabetes Reports. 2010;10(6):422-35.
17. **Otte-Trojel** T, de Bont A, Rundall TG, van de Klundert J. How outcomes are achieved through patient portals: A realist review. Journal of the American Medical Informatics Association. 2014;21(4):751-7.
18. **Price** M, Bellwood P, Kitson N, Davies I, Weber J, Lau F. Conditions potentially sensitive to a personal health record (PHR) intervention, a systematic review. BMC Med Inform Decis Mak. 2015;15:32.
19. **Stellefson** M, Chaney B, Barry AE, Chavarria E, Tennant B, Walsh-Childers K, et al. Web 2.0 chronic disease self-management for older adults: a systematic review. Journal of medical Internet research. 2013;15(2):e35.
20. **Tao** D, Or CKL. Effects of self-management health information technology on glycaemic control for patients with diabetes: a meta-analysis of randomized controlled trials. Journal of Telemedicine and Telecare. 2013;19(3):133-43.
21. **Tulu** B, Trudel J, Strong DM, Johnson SA, Sundaresan D, Garber L. Patient Portals: An underused resource for improving patient engagement. Chest. 2016;149(1):272-7.
22. **Turner** K, Klaman SL, Shea CM. Personal health records for people living with HIV: a review. AIDS Care - Psychological and Socio-Medical Aspects of AIDS/HIV. 2016;28(9):1181-7.
23. **Vimalananda** VG, Gupte G, Seraj SM, Orlander J, Berlowitz D, Fincke BG, et al. Electronic consultations (e-consults) to improve access to specialty care: A systematic review and narrative synthesis. Journal of Telemedicine and Telecare. 2015;21(6):323-30.
