# Supplementary material for: Methods to Evaluate the Effects of Internet-Based Digital Health Interventions for Citizens: Systematic Review of Reviews
Source: J Med Internet Res. 2018 Jun 7;20(6):e10202. doi: 10.2196/10202 (PMC6013714; doi:10.2196/10202)
Supplement: Multimedia Appendix 4 [file jmir_v20i6e10202_app4.pdf]

1. **Archer** N, Fevrier-Thomas U, Lokker C, McKibbon KA, Straus SE. Personal health records: A scoping review. Journal of the American Medical Informatics Association. 2011;18(4):515-22.
2. **Benhamou** PY, Muller M, Lablanche S, Debaty I. Telemedicine in the management of diabetic patients: Current developments and conditions for success. European Research in Telemedicine. 2013;2(1):23-8.
3. **Brunetti** ND, Scalvini S, Acquistapace F, Parati G, Volterrani M, Fedele F, et al. Telemedicine for cardiovascular disease continuum: A position paper from the Italian Society of Cardiology Working Group on Telecardiology and Informatics. International Journal of Cardiology. 2015;184(1):452-8.
4. **Cahill** JE, Gilbert MR, Armstrong TS. Personal health records as portal to the electronic medical record. J Neurooncol. 2014;117(1):1-6.
5. **Cox** CE, Randall Curtis J. Using technology to create a more humanistic approach to integrating palliative care into the intensive care unit C. American Journal of Respiratory and Critical Care Medicine. 2016;193(3):242-50.
6. **Gremeaux** V, Coudeyre E. The Internet and the therapeutic education of patients: A systematic review of the literature. Ann Phys Rehabil Med. 2010;53(10):669-92.
7. **Health Quality Ontario**. Electronic tools for health information exchange: an evidence-based analysis. Ontario health technology assessment series. 2013;13(11):1-76.
8. **Hoerbst** A, Ammenwerth E. Electronic Health Records A Systematic Review on Quality Requirements. Methods of Information in Medicine. 2010;49(4):320-36.
9. **Kazmi** Z. Effects of exam room EHR use on doctor-patient communication: a systematic literature review. Inform Prim Care. 2013;21(1):30-9.
10. **Ko** H, Turner T, Jones C, Hill C. Patient-held medical records for patients with chronic disease: a systematic review. Quality & safety in health care. 2010;19(5):e41.
11. **Kraschnewski** JL, Gabbay RA. Role of health information technologies in the patient-centered medical home. Journal of Diabetes Science and Technology. 2013;7(5):1376-85.
12. **Lachance** P, Villeneuve PM, Wilson FP, Selby NM, Featherstone R, Rewa O, et al. Impact of e-alert for detection of acute kidney injury on processes of care and outcomes: Protocol for a systematic review and meta-analysis. BMJ Open. 2016;6(5).
13. **Lau** F, Price M, Boyd J, Partridge C, Bell H, Raworth R. Impact of electronic medical record on physician practice in office settings: a systematic review. BMC Med Inform Decis Mak. 2012;12:10.
14. **LeBlanc** TW, Back AL, Danis M, Abernethy AP. Electronic Health Records (EHRs) in the oncology clinic: How clinician interaction with EHRs can improve communication with the patient. Journal of Oncology Practice. 2014;10(5):317-21.
15. **Mold** F, de Lusignan S, Sheikh A, Majeed A, Wyatt JC, Quinn T, et al. Patients' online access to their electronic health records and linked online services: a systematic review in primary care.
16. **Otte-Trojel** T, de Bont A, Rundall TG, van de Klundert J. What do we know about developing patient portals? A systematic literature review. Journal of the American Medical Informatics Association. 2016;23(e1):e162-e8.
17. **Prey** JE, Woollen J, Wilcox L, Sackeim AD, Hripcsak G, Bakken S, et al. Patient engagement in the inpatient setting: A systematic review. Journal of the American Medical Informatics Association. 2014;21(4):742-50.
18. **Turvey** CL, Roberts LJ. Recent developments in the use of online resources and mobile technologies to support mental health care. International Review of Psychiatry. 2015;27(6):547-57.
19. **Wittenberg**-Lyles E, Parker Oliver D, Demiris G, Swarz J, Rendo M. YouTube as a tool for pain management with informal caregivers of cancer patients: A systematic review. Journal of Pain and Symptom Management. 2014;48(6):1200-10.
